# The Performance of Loghman-Hakim Drug and Poison Information Center from 2006 to 2008

**Published:** 2011

**Authors:** Shahin Shadnia, Kambiz Soltaninejad, Farrokh Sohrabi, Manijeh Rezvani, Behjat Barari, Mohammad Abdollahi

**Affiliations:** a *Loghman-Hakim Hospital Poison Center, Faculty of Medicine and Toxicological Research Center, Shahid Beheshti University of Medical Sciences, Tehran, Iran.*; b *Department of Forensic Toxicology, Legal Medicine Research Center, Legal Medicine Organization, Tehran, Iran.*; c *Loghman-Hakim Drug and Poison Information Center, Shahid Beheshti University of Medical Sciences, Tehran, Iran.*; d *Faculty of Medicine and Pharmaceutical Sciences Research Center, Tehran University of Medical Sciences, Tehran, Iran.*

**Keywords:** Drug and Poison Information Center, Iran, Loghman-Hakim Hospital, Patient, DPICs

## Abstract

Drug and poison information centers have a critical role in fulfillment of rational drug use programs. The Loghman-Hakim Drug and Poison Information Center (LHDPIC) has been established in 2006. The main mission of this center is to provide accurate, unbiased and up-to-date information on medications and poisons for the health care team and the public. This center has received more than 9000 telephone calls since its establishment. The aim of this study was to evaluate the recorded queries in the LHDPIC in the past 3 years.

A descriptive analysis was conducted on all recorded inquiries to the center from March 2006 to March 2008. Data such as patient age and sex, identity of the callers, question categories and information resources were obtained from the recorded calls and were analyzed.

During the period of evaluation, a total of 9694 telephone calls were recorded. The patients’ age ranged between 18-40 years old (49.42%) containing 61% female and 39% male. Most of the recorded calls were from patients’ relatives (49%) and then the patients (45.2%) themselves. The most frequent questions were about drug indications (24%), adverse drug reactions (20.14%) and drug evaluation (17.64%). Antidepressants (12.42%), antimicrobials (12%) and analgesics (11.17%) were the most frequent drug classes that were inquired.

The LHDPIC has an important role for providing of drug information for the lay public, but more efforts are still needed to encourage health care professionals to utilize services provided by this center.

## Introduction

Drug and poison information centers (DPICs) have an important role both in supporting rational drug use and collecting data on the prevalence of adverse drug reactions and poisoning. Existence of DPICs improves national drug policy and its standardized indicators in the country. Considering constant growth of medications that are approved for clinical use, there is a need to update the health care professionals in determining dosages, uses and other medication-related issues. On the other hand, physicians and pharmacists have limited the access to the resources of information about drugs and chemicals. For these reasons, DPICs were established with the primary goal of providing accurate and timely drug information to those in urgent need of such information (1).The first national DPIC in Iran was established in Tehran in early 1995, as a part of food and drug organization of Iran under supervision of the Ministry of Health and Medical Education (MOH) as the national DPIC. Since establishment, it has had an important role for policy making in the dissemination of DPICs in the country. Then, by co-operation of the national DPIC, other DPICs were established countrywide in most of the provinces and the staffs were trained by the national DPIC to learn how to work in a DPIC. At the present, there are 29 active DPICs in Iran working as a network under the supervision of the Medical Universities and MOH (2, 3). Loghman-Hakim DPIC (LHDPIC) is one of them which is established in Loghman Hakim-Hospital Poison Center as a referral center for poisoning, in 2006. The center provides information and advice on medications and poisoning 12 h a day, 7 days a week, from 7 pm to 7 am, but on holidays, the service is 24 h a day. The center has a clinical toxicologist as a director and a toxicologist as a scientific consultant. In the present study, all the recorded queries in the LHDPIC from 2006 to 2008 have been evaluated.

## Experimental

*Methods*

A special form was used to obtain information regarding every call received by the LHDPIC from March 21, 2006 to March 21, 2008. Collected data included details of the caller, the date and time of the call, the patient›s demographics, nature of enquiry, the chemical or medication which the call focused on and the information resources used to support information provision. Data from this form was extracted and then analyzed statistically using MS-Excel 2007.

## Results and Discussion

**Figure 1:**
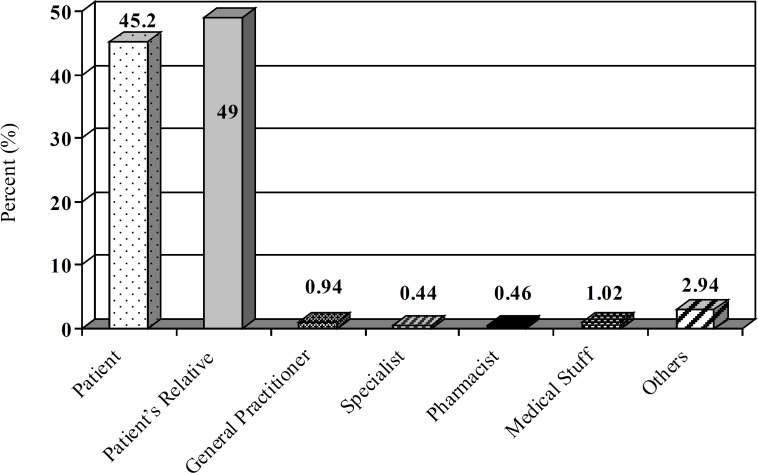
Distribution of callers to Loghman-Hakim Drug and Poison Information Center from March 2006 to March 2008

**Figure 2:**
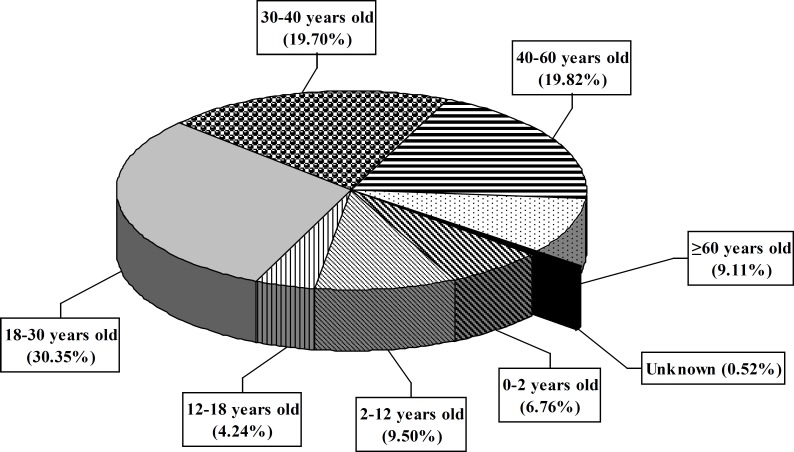
Distribution of patients in Loghman-Hakim Drug and Poison Information Center according to age groups from March 2006 to March 2008

A total of 9694 calls were received during this period. The center received 2671, 2576 and 4447 inquiries during 2006, 2007 and 2008 years, respectively. Most calls were made by the patients’ relatives (49%) and then the patients (45.2%) followed by medical staff (2.86%; Figure 1). All calls were made from different places in Tehran. The gender of the callers showed that 39% were males and 61% were females. The majority of patients (30.35%) were 18-30 years old (Figure 2). Response time was recorded and about 98% of queries were answered within 30 min. All queries were received through telephone and the mode of replay was oral. Most of questions were about drug indications and adverse drug reactions (ADR) (23.9% and 20.14%, respectively) (Table 1).

**Table 1:** The distribution of records according to the type of questions

**Request Classification **	**2006 **	**2007 **	**2008 **	**Total (%)**
**Drug Evaluation and Identification **	529	489	863	1881 (17.64%)
**Drug Indication **	789	711	1049	2549 (23.9%)
**Drug Administration **	220	497	692	1409 (13.21)
**Adverse Drug Reaction (ADR)**	695	550	903	2148 (20.14%)
**Drug Dosage **	162	127	357	646 (6.06%)
**Drug Interaction **	165	168	225	558 (5.23%)
**Drug in Pregnancy **	83	116	275	474 (4.44%)
**Drug in Lactation **	57	91	98	246 (2.31%)
**Poisoning and Toxicology **	115	70	152	337 (3.16%)
**Drug Abuse **	11	9	17	37 (0.35%)
**Drug Contraindication and Caution **	49	32	91	172 (1.61%)
**Pharmacology **	8	3	12	23 (0.22%)
**Storage, Stability and Compatibility **	14	12	47	73 (0.68%)
**Drug Therapy and Comparison **	15	29	68	112 (1.05%)

Regarding compound name, the majority of queries were about antidepressants (12.42%), followed by antimicrobials (12%) and analgesics (11.17%) (Table 2). 

**Table 2:** The distribution of records according to the classes of drugs

**Class of Drug**	**2006**	**2007**	**2008**	**Total (%)**
**Antimicrobial**	317	394	792	1503 (12%)
**Antiseptic**	103	13	30	146 (1.16%)
**Analgesics and Anesthetic**	387	379	634	1400 (11.17%)
**Sedative**	321	178	271	770 (6.15%)
**Anticonvulsant**	81	80	167	328 (2.62%)
**Antidepressant**	400	433	723	1556 (12.42%)
**Antipsychotic**	42	80	112	234 (1.87%)
**Antiparkinsonism**	23	17	34	74 (0.6%)
**Digitalis**	22	14	45	81 (0.65%)
**Antihypertensive**	159	180	319	658 (5.25%)
**Antiarrythmic**	38	54	82	174 (1.39%)
**Antianginal (heart attack)**	18	68	69	155 (1.24%)
**Antidiabetic**	26	57	134	217 (1.73%)
**Antihyperlipidemic**	37	37	108	182 (1.45%)
**Gastrointestinal**	264	411	570	1245 (9.94%)
**Dermatologics**	60	43	100	203 (1.62%)
**Hair Products**	23	23	54	100 (0.8%)
**Cosmetics**	13	10	31	54 (0.43%)
**Diagnostics**	14	10	36	60 (0.48%)
**Aphrodisiactic Drugs**	338	165	332	835 (6.65%)
**Hormones**	106	92	162	360 (2.87%)
**Complementary/Alternative Medicine Agents**	29	36	62	127 (1%)
**Vaccines**	17	25	60	102 (0.8%)
**Otic-Ophthalmic**	36	27	79	142 (1.13%)
**Oral products**	113	18	40	71 (0.57%)
**Inhalants**	44	42	68	154 (1.23%)
**Nasal products**	47	21	70	138 (1.1%)
**Vitamins and Minerals**	130	116	268	514 (4.1%)
**Total Parenteral Nutrition(TPN)**	13	18	31	62 (0.5%)
**Volume Expander/Blood Agents**	56	77	135	268 (2.14%)
**Immunological Agents**	13	16	32	61 (0.49%)
**Herbal Products**	28	12	47	87 (0.7%)
**Musculoskeletal Drugs**	38	65	150	253 (2.02%)
**Antigout**	14	12	36	62 (0.5%)
**Anti-rheumatoid**	16	37	59	112 (0.89%)
**Antidote**	11	11	20	42 (0.34%)

The poisoning and toxicology information composed only 3.16% of the received queries. The majority of poisoning was induced by pharmaceuticals (70.04%). Sedative-hypnotics especially benzodiazepines and antidepressants were the major causes of drug poisonings. Other causes of poisoning were chemicals (22%), herbs (3.62%), foods (2.65%) and bites, especially scorpions and bees (1.69%). The questions were answered by the use of information resources such as Micromedex® Healthcare Series (USA, 72%), Iranpharma® (Iran, 24.48%), Drugs in Pregnancy and Lactation (Briggs, 1.90%), Internet search (0.75%), Martindale (the Complete Drug Reference, 0.71%) and others like Goodman and Gilman’s the Pharmacological Basis of Therapeutics, National Formulary of Iran (NFI), Harrison’s Principles of Internal Medicine, Merck Index, Haddad and Winchester’s Clinical Management of Poisoning and Drug Overdose, Ellenhorn›s Medical Toxicology, Diagnosis and Treatment of Human Poisoning, and INCHEM (0.16%).

 Present data show that LHDPIC has provided information, advice and served as a guide for patient management and raised the community awareness regarding drug information. There was an increase in the numbers of calls in 2008 and this indicates the public awareness about the activity of the center. The patients’ relatives and females were the most frequent groups who called LHDPIC that is in accordance with previous report of national DPIC of Iran (4). This indicates more sensitivity and responsibility of patients’ relatives about disease status, prescribed drugs, and fate of their patients. The LHDPIC was created to provide unbiased up-to-date drug information to the public for the attainment of high compliance to drug therapy. The calls received from health care professionals at LHDPIC were too little. This finding is different from previous reports of other countries (5-10) that most probably return to our different work time. The LHDPIC’s work times are at non-official hours and it may be an explanation for few received calls to the center from health care professionals. Another reason may be related to the ignorance of the medical staff about DPIC’s services and abilities. Therefore, it is important that the DPIC’s services should be adequately communicated to health care providers through various media.

Most of questions were about drug indications and ADR. These findings are similar to previous reports from other countries and also our own country (4-10). It may be the result of public accessibility to the Internet and other media, which results in more awareness about drug-induced adverse reactions.The majority of questions were about antidepressants, followed by antimicrobials and analgesics. This finding is in accordance with a previous study in Iran and describes status and pattern of common drug usage at least in Tehran (4) and is in accordance with official reports about the pattern of drug usage in Iran (11).Despite the location of the center (inside a referral poison center) and epidemiology of poisoning in Tehran (12), the poisoning queries received at the center were low. This finding is similar to previous reports from Tehran (4, 13) and seems related to the inadequate general education of the public for calling a DPIC especially in early and pre-hospital phase of poisoning. However, the early phase of poisoning is an opportune time for the treatment of poisoning cases. DPIC has a crucial role for providing information about standard procedures for pre-hospital phase of acute poisoning especially for the lay public. Therefore, more public general education programs for the achievement to this goal are necessary.

The time between calling and answering in LHDPIC showed that most of the calls were answered immediately on the same day. It is attributed to the suitable training and experience of LHDPIC’s staff for using drug information resources. Micromedex® is one of the most useful drug information resources in our center. This is related to the comprehensive nature of this drug information resource and the ease of retrieval of relevant information for the drug information process. Other updated textbooks and specialized texts on medicine and pharmacy are still needed to maintain the drug information process. However, the staff at the center recognized that the use of electronic databases as a main resource for the drug information process is more suitable as supported with our previous report. 

*Conclusion and recommendation*

 The present study showed that the LHDPIC met its objectives of providing drug information for the public but attempts should be made to educate the public about calling DPIC in poisoning cases, especially in the pre-hospital phase. Continuous education of DPIC staff and the establishment of external quality assurance program for DPICs in national levels should be considered. 
